
JOURNAL INFORMATION
==============================
NLM Title Abbreviation: ScientificWorldJournal
Journal ID: ScientificWorldJournal
NLM Journal ID: 101131163
Journal ID: TSWJ

The Scientific World Journal

ISSN: 2356-6140
EISSN: 1537-744X
Publisher: Wiley

ARTICLE INFORMATION
==============================
PMCID: PMC6009342
PMID: 29973848
DOI: 10.1100/tsw.2002.165
Article ID: 272149
Article version: 1
Subjects: Editorial

Founding Editorial: Higher Level Brain Function

Posner Michael
Publication date: 2002
Electronic publication date: 2002 Mar 15
Volume: 2
First page: 738
Last page: 738
Received 2002 Feb 12; Accepted 2002 Feb 13
Copyright: Copyright © 2002 Michael Posner.
Copyright year: 2002
License: This is an open access article distributed under the Creative Commons Attribution License, which permits unrestricted use, distribution, and reproduction in any medium, provided the original work is properly cited.
License URL: https://creativecommons.org/licenses/by/3.0/

Keywords: cognition, brain lesions, genetics, pharmacology, rehabilitation

==============================
Founding Editorial
*TheScientificWorldJOURNAL* (2002) 2, 738
ISSN 1537-744X; DOI 10.1100/tsw.2002.165

TheScientificWorld
www.thescientificworld.com

### Founding Editorial: Higher Level Brain Function

Michael Posner
*Principal Editor, Higher Level Brain Function*

*Received February 12, 2002; Accepted February 13, 2002; Published March 15, 2002*

**KEY WORDS:** cognition, brain lesions, genetics, pharmacology, rehabilitation

**DOMAINS:** higher level brain function

With the rapid growth of Affective, Cognitive, Developmental, and Social Neuroscience, the domain of Higher Level Brain Function has become of central importance to understanding the human brain. In this Domain we welcome papers using neuroimaging, genetics, pharmacology, and other methods in studies both directly involving the human brain as well as nonhuman animals. These studies may elucidate how the brain relates to thoughts, feelings, and complex behaviour.

The Higher Level Brain Function Domain will be closely coordinated with the Cognition and other related Domains to ensure that all appropriate submissions are handled rapidly and effectively. As with other Domains, we hope to ensure that the Higher Level Brain Function Domain is a rapid communication environment for papers that have been thoroughly peer reviewed. While not all papers can be published, we would like to be prompt, clear, and helpful in dealing with each submission. We have a large and distinguished Editorial team working on this Domain that represents a range of research interests. We hope that this is reflected in the submissions that we receive.

**This article should be referenced as follows:**

Posner, M. (2002) Founding editorial: Higher Level Brain Function. *TheScientificWorldJOURNAL* **2**, 738.

Email: mip2003@med.cornell.edu
©2002 with author.
